# Case Report: Usefulness of transcutaneous bilirubin measurement for the early detection of severe hyperbilirubinemia due to late-onset hemolysis in an extremely low birth weight infant

**DOI:** 10.3389/fped.2026.1926166

**Published:** 2026-08-28

**Authors:** Kae Tanaka, Takeshi Utsunomiya, Kurita Nakayama, Kana Tsuneishi, Tsubasa Koda, Masumi Okuda, Yasuhiro Takeshima

**Affiliations:** Department of Pediatrics, School of Medicine, Hyogo Medical University, Nishinomiya, Hyogo, Japan

**Keywords:** case report, extremely low-birth-weight infants, late-onset hemolysis, neonatal jaundice, total serum bilirubin, transcutaneous bilirubin

## Abstract

**Background:**

Late-onset hemolysis is a rare but potentially severe complication in extremely preterm infants and may cause rapid increases in bilirubin levels requiring intensive treatment, including exchange transfusion. Phototherapy for neonatal jaundice is guided by total serum bilirubin (TSB) and unbound bilirubin (UB) levels; however, in extremely low-birth-weight infants (ELBWIs), defined as neonates with a birth weight of less than 1,000 g, bilirubin neurotoxicity may occur at lower thresholds, and severe hyperbilirubinemia can develop unexpectedly beyond the acute neonatal phase. Therefore, early recognition of progressive hyperbilirubinemia is essential in this high-risk population.

**Case presentation:**

We report the case of a male extremely low-birth-weight infant born at 23 weeks and 1 day of gestation with a birth weight of 598 g who developed severe hyperbilirubinemia due to late-onset hemolysis on day of life (DOL) 15. Serial transcutaneous bilirubin (TcB) measurements revealed a rapid increase in bilirubin levels from 5.5 mg/dL to 14.3 mg/dL between DOL 14 and 15, prompting further evaluation. At DOL 15, total serum bilirubin (TSB) and unbound bilirubin (UB) levels were 18.0 mg/dL and 2.00 μg/dL, respectively, exceeding the exchange transfusion threshold. High-intensity phototherapy, albumin administration, and subsequent exchange transfusion were performed. Laboratory findings, including elevated carboxyhemoglobin and methemoglobin levels, supported the diagnosis of late-onset hemolytic jaundice. The infant recovered without recurrence of jaundice or neurological abnormalities during follow-up.

**Conclusion:**

Late-onset hemolysis can occur even in extremely immature infants and may lead to severe hyperbilirubinemia requiring exchange transfusion. Systematic bilirubin surveillance, including non-invasive monitoring approaches such as TcB, may help reduce diagnostic delays and mitigate the risk of bilirubin neurotoxicity in high-risk preterm infants. In this case, serial TcB measurements contributed to the early detection of rapidly increasing bilirubin levels and facilitated timely intervention.

## 1 Introduction

Late-onset hemolysis is a recognized but potentially severe complication in very low birth weight and extremely preterm infants and may result in rapidly progressive hyperbilirubinemia requiring intensive treatment, including exchange transfusion (1). Several cases of transient, unexplained hemolysis after the acute phase of illness have been reported in very-low-birth-weight infants (VLBWIs), characterized by sudden increases in bilirubin levels, elevated methemoglobin (MetHb) levels, Heinz body formation, and the need for intensive management (1). Because late-onset hemolysis can cause rapidly progressive hyperbilirubinemia, early recognition is essential in this high-risk population. Unbound bilirubin (UB), which is associated with bilirubin neurotoxicity in preterm infants, may provide important information for assessing the risk of bilirubin encephalopathy (2). However, clinical recognition of late-onset hemolysis remains challenging, particularly in extremely low-birth-weight infants (ELBWIs), because it develops after the acute neonatal period without specific early clinical signs, and routine bilirubin surveillance is not universally performed beyond the initial postnatal period (1).

Some patients with bilirubin encephalopathy do not exhibit marked elevations in total serum bilirubin (TSB), and peak bilirubin neurotoxicity may occur beyond the early neonatal period (2). In Japan, recently established criteria for managing hyperbilirubinemia (3) emphasize assessment based on corrected day of life and incorporate both UB and TSB measurements. However, UB measurement is available in only 79 of 172 participating facilities (45.9%) in Japan (4), resulting in variability in the management of neonatal jaundice. Therefore, continuous monitoring of bilirubin levels is important, although frequent blood sampling is challenging in extremely preterm infants.

Transcutaneous bilirubin (TcB) measurement is a non-invasive method for monitoring bilirubin levels and may reduce the need for blood sampling (5). In preterm infants, TcB measurements have been reported to correlate well with TSB and UB levels (6), and the sternum or upper back has been identified as a reliable site for taking measurements (7). However, TcB monitoring has not been universally implemented in neonatal intensive care units in Japan (8).

We report a case of an ELBWI born at 23 weeks of gestation who developed severe hyperbilirubinemia due to late-onset hemolysis on day of life (DOL) 15, requiring exchange transfusion. Serial TcB monitoring enabled early recognition of rapidly increasing bilirubin levels and facilitated prompt evaluation and treatment. This case highlights the potential role of serial TcB monitoring in the early recognition of late-onset hemolysis in extremely preterm infants.

## 2 Case presentation

The patient was a male infant born at 23 weeks and 1 day of gestation via emergency cesarean section because of fetal bradycardia. His birth weight was 598 g. APGAR scores at 1, 5, and 10 min were 3, 7, and 8, respectively. Surfactant was administered shortly after birth, and a prophylactic dose of indomethacin was administered for patent ductus arteriosus (PDA) on DOL 1, followed by two therapeutic doses on DOL 6 and 7. Ampicillin and gentamicin were administered from DOL 0 to 2, and intravenous immunoglobulin (IVIg, 500 mg/kg) was administered on DOL 1 as prophylactic therapy for severe infection, in compliance with our institutional protocols. The initial hemoglobin level at admission was 14.4 g/dL. Due to anemia of prematurity and phlebotomy-associated blood loss, packed red blood cell transfusions were administered on DOL 4 and DOL 5. Hydrocortisone was administered from DOL 11 to DOL 20 because of increasing oxygen requirements. Phototherapy was administered for hyperbilirubinemia on DOL 2, 6, and 10. Vitamin K was administered intravenously on DOL 10, as part of routine Japanese care guidelines (9). Between DOL 14 and DOL 15, TcB levels rapidly increased from 5.5 mg/dL to 14.3 mg/dL. At our facility, daily TcB measurements are performed, taking into account their skin condition and overall clinical status. Also, TSB is measured in infants with a corrected gestational age of <25 weeks and 6 days when the TcB level exceeds 6 mg/dL.

**Figure 1:**
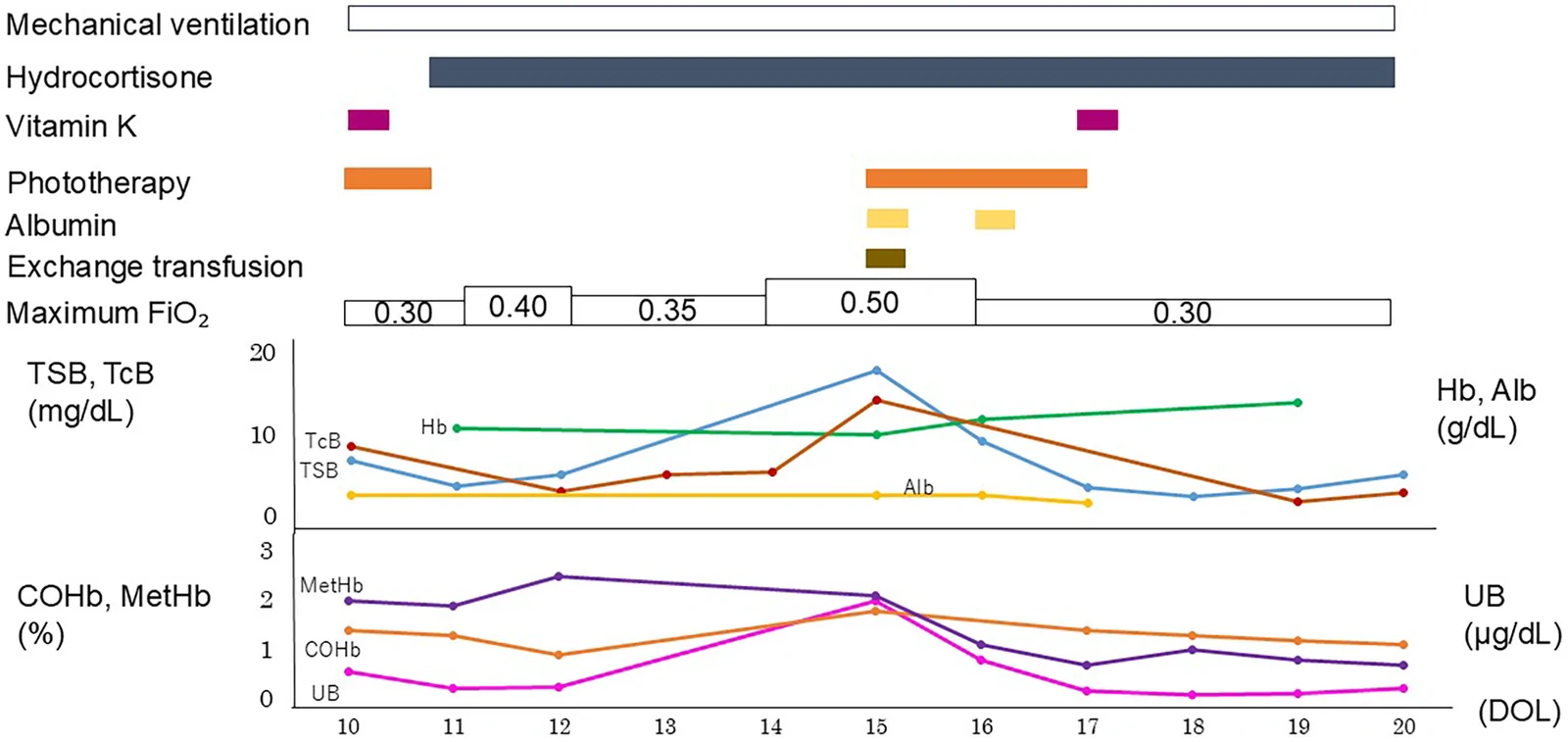
Clinical progression in this case. On day of life (DOL) 15, the levels of transcutaneous bilirubin (TcB), total serum bilirubin (TSB), carboxyhemoglobin (COHb), and unbound bilirubin (UB) peaked, whereas those of hemoglobin remained stable. UB, unbound bilirubin, MetHb, methemoglobin, COHb, carboxyhemoglobin, DOL, day of life, FiO_2_, fraction of inspired oxygen.

On DOL 15, while receiving high-frequency ventilation (FiO₂ 0.50, mean airway pressure 14 cm H₂O, and high-frequency tidal volume 1.5 mL), the patient’s SpO₂ was 90%, heart rate 140 beats per minute, blood pressure 61/33 mmHg, and axillary temperature 37.2 °C. Physical examination revealed no significant abnormalities. Significant laboratory findings included mixed acidosis with elevated lactate dehydrogenase, alkaline phosphatase, carboxyhemoglobin (COHb) and MetHb levels (Table 1; Figure 1), an increased white cell count and a reduced hemoglobin level of 10 g/dL (a drop from 10.9 g/dL on DOL 11). The TSB level was 18.0 mg/dL, and the UB level was 2.00 µg/dL, measured using a Pharmaceuticals and Medical Devices Agency-approved UB analyzer (Arrows, Osaka, Japan); both values exceeded the exchange transfusion threshold defined by Morioka’s criteria (TSB/UB: 13 mg/dL/0.8 µg/dL) (3). Results for C-reactive protein, direct/indirect antiglobulin test (DAT/IAT), chest radiography, echocardiogram, and abdominal ultrasound were unremarkable. There was no documented family history of hemolytic jaundice or glucose-6-phosphate dehydrogenase deficiency.

**Table 1:** Laboratory tests on day of life 15.

Laboratory test	Value	Unit	Laboratory test	Value	Unit
pH	7.247		Direct bilirubin	0.9	mg/dL
pCO_2_	52.3	mmHg	AST	15	U/L
HCO_3_^−^	19.7	mmol/L	ALT	2	U/L
Glucose	142	mg/dL	LDH	443	U/L
Lactate	2.4	mmol/L	ALP	674	U/L
White blood cell	19,340	/μL	BUN	30	mg/dL
Hemoglobin	10.0	g/dL	Creatinine	0.76	mg/dL
Hematocrit	29.6	%	Sodium	140	mmol/L
Platelet	40.1	×10^4^/μL	Potassium	5.1	mmol/L
Albumin	2.5	g/dL	Chloride	109	mmol/L
Total serum bilirubin	15.6	mg/dL	C-reactive protein	0.00	mg/dL

Total serum bilirubin was measured using the colorimetric method.

WBC, white blood cell; AST, aspartate aminotransferase; ALT, alanine aminotransferase; LDH, lactate dehydrogenase; ALP, alkaline phosphatase; BUN, blood urine nitrogen.

The MetHb level was 2.0% on DOL 10 and increased to 2.5% on DOL 12. On DOL 15, there was a concurrent increase in TSB and UB levels, along with elevated TcB levels. Upon confirmation of elevated TSB (18.0 mg/dL) and UB (2.00 μg/dL) levels, high-intensity phototherapy and albumin administration were initiated because the UB level exceeded the exchange transfusion threshold defined by Morioka’s criteria. In Japan, albumin administration is considered when UB levels exceed the exchange transfusion threshold, as albumin binds unconjugated bilirubin and may reduce the risk of bilirubin neurotoxicity (3, 10). Exchange transfusion was performed after the necessary preparations were completed. After exchange transfusion, the TSB and UB levels had decreased to 9.4 mg/dL and 0.70 µg/dL, respectively. As both values had improved to below the exchange transfusion threshold defined by Morioka’s criteria (TSB/UB: 13 mg/dL/0.8 µg/dL), the exchange transfusion was discontinued. No recurrence of jaundice was observed. Based on the clinical progression and laboratory findings, this case was consistent with late-onset hemolytic jaundice in an ELBWI. Brain MRI at 40 weeks of corrected age revealed a mild upper brainstem hemorrhage, with no abnormal findings in the globus pallidus. At the time of reporting, the patient was 8 months old (with a corrected age of 4 months from the expected birth date) and exhibited no apparent neurological abnormalities or developmental delays.

## 3 Discussion

Since 1996, several VLBWIs in Japan have exhibited transient, unexplained hemolysis following the acute phase of illness (1). Similar clinical courses have occasionally been reported outside Japan; however, reports of late-onset hemolysis in VLBWIs remain limited internationally (11). A nationwide survey in Japan reported that late-onset hemolysis was characterized by onset at 1–4 weeks of age, elevated MetHb levels or Heinz body formation, transient hemolysis, and, in some cases, the need for intensive treatment including exchange transfusion (1). These findings suggest that late-onset hemolysis may be an underrecognized condition in extremely preterm infants rather than a phenomenon limited to a specific region. Our patient shared many features with previously reported cases, including extremely low birth weight, onset after the acute phase of illness, elevated MetHb levels, and a transient clinical course. However, this case is notable because serial TcB measurements facilitated the early recognition of rapidly increasing bilirubin levels and prompted timely evaluation in this extremely preterm infant developing severe hyperbilirubinemia due to late-onset hemolysis and requiring exchange transfusion.

In this patient, numerous aspects of the clinical course, signs, and laboratory findings at onset were consistent with those reported previously (1), including ELBW, surfactant administration for RDS, indomethacin treatment for PDA, acute development of jaundice on DOL 15 requiring phototherapy and ET, elevated COHb levels indicating increased heme catabolism, elevated MetHb levels, absence of a family history of hematologic disorders, and no exposure to medications known to induce MetHb elevation. IVIg-associated hemolysis has also been reported to occur several days after administration, particularly in patients receiving high-dose IVIg therapy (12). The interval between IVIg administration and the onset of hemolysis in our patient was 14 days. The patient had also received packed red blood cell transfusions on DOL 4 and DOL 5; however, hemolysis occurred 10 days later, making a transfusion-related hemolytic reaction less likely. In addition, the clinical features observed in this case, including elevated COHb and MetHb levels and the transient course of hemolysis, were consistent with previously reported late-onset hemolysis in VLBWIs (1). The elevated MetHb levels observed from DOL 10 may have reflected increased oxidative stress associated with extreme prematurity and intensive respiratory management. Given that extremely preterm infants have immature antioxidant systems, they may be more susceptible to oxidative injury of erythrocytes. However, the exact mechanism underlying the persistent elevation of MetHb levels in this case remains unclear. Also, although prophylactic IVIg administration for infection prevention in extremely preterm infants has not been shown to provide significant clinical benefits according to a Cochrane review (13), it continues to be used as a routine practice in some institutions in Japan. The patient had a transient clinical course without recurrence of hemolysis. In this case, the progression of anemia was relatively slow compared with previously reported cases. Although the reticulocyte count was monitored, reticulocytosis was not prominent at the onset of hyperbilirubinemia. However, the elevated COHb level, which reflects increased heme catabolism, supported the presence of hemolysis. This patient did not exhibit elevated direct bilirubin levels. When direct bilirubin levels are elevated, UB may appear pseudo-elevated (14). In our patient, UB levels were elevated, with normal direct bilirubin levels.

In our patient, TcB measurements were performed as part of the diagnostic evaluation. Serial TcB measurements were useful for detecting the rapid increase in bilirubin levels. To date, TcB measurement has been reported as a diagnostic trigger in only one previous case (15). TcB levels were consistently elevated in this patient. In addition to absolute bilirubin levels, the rate of rise (RoR) of bilirubin may provide an important clue for identifying rapidly progressive hyperbilirubinemia and prompting further evaluation for hemolysis. In this case, daily TcB measurements enabled detection of a rapid increase in bilirubin levels, instigating further evaluation and timely intervention. At our facility, TcB was measured using a JM105 device (Konica Minolta, Tokyo, Japan). TcB measurements correlate well with TSB values and are reliable for evaluating preterm infants with hyperbilirubinemia (16). Although TcB is not used at all facilities in Japan (4), our case suggests that TcB measurements may improve the management of preterm infants both in Japan and in other countries.

This study has several limitations. First, Heinz body evaluation could not be performed because the necessary testing facilities are unavailable at our institution. Second, given this is a single case report, the generalizability of our findings is limited. Further studies are needed to determine the clinical utility of serial TcB measurements for detecting late-onset severe hyperbilirubinemia in extremely preterm infants.

## 4 Conclusion

Late-onset hemolysis can occur even in extremely preterm infants and may result in rapidly progressive hyperbilirubinemia requiring exchange transfusion. Serial TcB measurements contributed to the early recognition of increasing bilirubin levels and facilitated timely evaluation and intervention. TcB may help to prevent diagnostic delays and mitigate the risk of bilirubin neurotoxicity in high-risk preterm infants.

## Data Availability

The original contributions presented in the study are included in the article/Supplementary Material, further inquiries can be directed to the corresponding author.
