# Supplementary material for: Case Report: Usefulness of transcutaneous bilirubin measurement for the early detection of severe hyperbilirubinemia due to late-onset hemolysis in an extremely low birth weight infant
Source: Front Pediatr. 2026 Aug 28;14:1926166. doi: 10.3389/fped.2026.1926166 (PMC13561966; doi:10.3389/fped.2026.1926166)
Supplement: Supplementary file 1 [file Supplementaryfile1.docx]

# CARE Checklist of information to include when writing a case report

**Topic	Item	Checklist item description	Reported on Line**

**Title	1**	The diagnosis or intervention of primary focus followed by the words “case report” . . . . . . . . . . . . . . . . . .	Title

**Key Words	2**	2 to 5 key words that identify diagnoses or interventions in this case report, including "case report"	Key words

Described 5–8 keywords in compliance with the journal guidelines.

**Abstract**

**(no references)**

**3a**	Introduction: What is unique about this case and what does it add to the scientific literature?	Abstract

**3b**	Main symptoms and/or important clinical findings . . . . . . . . . . . . . . . . . . . . . . . . . . . . . . . . . . . . . . . . . . . . . . . . . . .	Abstract

**3c**	The main diagnoses, therapeutic interventions, and outcomes	Abstract

**3d**	Conclusion—What is the main “take-away” lesson(s) from this case?	Abstract

**Introduction	4**	One or two paragraphs summarizing why this case is unique (**may include references**)	Introduction

**Patient Information	5a**	De-identified patient specific information	Case Presentation

**5b**	Primary concerns and symptoms of the patient	Case Presentation

**5c**	Medical, family, and psycho-social history including relevant genetic information	Case Presentation

**5d**	Relevant past interventions with outcomes	Case Presentation

**Clinical Findings**

**Timeline**

**Diagnostic Assessment**

**Therapeutic Intervention**

**Follow-up and Outcomes**

6 Describe significant physical examination (PE) and important clinical findings	Case presentation
7 Historical and current information from this episode of care organized as a timeline	Figure１

**8a**	Diagnostic testing (such as PE, laboratory testing, imaging, surveys).	Case presentation

**8b**	Diagnostic challenges (such as access to testing, financial, or cultural)	Case presentation

**8c**	Diagnosis (including other diagnoses considered)	Case presentation

**8d**	Prognosis (such as staging in oncology) where applicable

**9a**	Types of therapeutic intervention (such as pharmacologic, surgical, preventive, self-care) . . . . . . . . . . . .	Case presentation

**9b**	Administration of therapeutic intervention (such as dosage, strength, duration)	Case presentation

**9c**	Changes in therapeutic intervention (with rationale)	Case presentation

**10a**	Clinician and patient-assessed outcomes (if available)	Case presentation

**10b**	Important follow-up diagnostic and other test results	Case presentation

**10c**	Intervention adherence and tolerability (How was this assessed?)	Case presentation

**10d**	Adverse and unanticipated events	Case presentation

**Discussion	11a**	A scientific discussion of the strengths AND limitations associated with this case report	Discussion

**11b**	Discussion of the relevant medical literature **with references**	Discussion

**11c**	The scientific rationale for any conclusions (including assessment of possible causes)	Discussion

**11d**	The primary “take-away” lessons of this case report (without references) in a one paragraph conclusion	Conclusion

**Patient Perspective	12**	The patient should share their perspective in one to two paragraphs on the treatment(s) they received . . . .	Patient Perspective

**Informed Consent	13**	Did the patient give informed consent? Please provide if requested . . . . . . . . . . . . . . . . . . . . . . . . . . . . . . . . . . . . . .	**Yes ☑** **No**
